# Mutations in the BRCT binding site of BRCA1 result in hyper-recombination

**DOI:** 10.18632/aging.100325

**Published:** 2011-05-08

**Authors:** Seth M. Dever, Sarah E. Golding, Elizabeth Rosenberg, Bret R. Adams, Michael O. Idowu, John M. Quillin, Nicholas Valerie, Bo Xu, Lawrence F. Povirk, Kristoffer Valerie

**Affiliations:** ^1^ Department of Radiation Oncology, Virginia Commonwealth University, Richmond, VA 23298, USA; ^2^ Department of Biochemistry and Molecular Biology, Virginia Commonwealth University, Richmond, VA 23298, USA; ^3^ Department of Pathology, Virginia Commonwealth University, Richmond, VA 23298, USA; ^4^ Department of Human and Molecular Genetics, Virginia Commonwealth University, Richmond, VA 23298, USA; ^5^ Department Pharmacology and Toxicology, Virginia Commonwealth University, Richmond, VA 23298, USA; ^6^ Massey Cancer Center, Virginia Commonwealth University, Richmond, VA 23298, USA; ^7^ Department of Radiation Oncology, The Methodist Hospital, Houston, TX 77030, USA

**Keywords:** DNA repair, DNA damage, ubiquitination, radiation, PML bodies, breast cancer

## Abstract

We introduced a K1702M mutation in the BRCA1 BRCT domain known to prevent the binding of proteins harboring pS-X-X-F motifs such as Abraxas-RAP80, BRIP1, and CtIP. Surprisingly, rather than impairing homologous recombination repair (HRR), expression of K1702M resulted in hyper-recombination coinciding with an accumulation of cells in S-G2 and no effect on nonhomologous end-joining. These cells also showed increased RAD51 and RPA nuclear staining. More pronounced effects were seen with a naturally occurring BRCT mutant (M1775R) that also produced elevated levels of ssDNA, in part co-localizing with RPA, in line with excessive DNA resection. M1775R induced unusual, thread-like promyelocytic leukemia (PML) nuclear bodies and clustered RPA foci rather than the typical juxtaposed RPA-PML foci seen with wild-type BRCA1. Interestingly, K1702M hyper-recombination diminished with a second mutation in the BRCA1 RING domain (I26A) known to reduce BRCA1 ubiquitin-ligase activity. These *in vitro* findings correlated with elevated nuclear RAD51 and RPA staining of breast cancer tissue from a patient with the M1775R mutation. Altogether, the disruption of BRCA1 (BRCT)-pS-X-X-F protein binding results in ubiquitination-dependent hyper-recombination via excessive DNA resection and the appearance of atypical PML-NBs. Thus, certain BRCA1 mutations that cause hyper-recombination instead of reduced DSB repair might lead to breast cancer.

## INTRODUCTION

Mutations in DNA repair genes, including breast cancer susceptibility 1 (*BRCA1*), are closely linked to the development of cancer. While mutations in *BRCA1* are known to significantly increase the chances of developing breast and ovarian cancers, the mechanism behind this predisposition is not fully understood. Cells defective in BRCA1 are compromised in both major forms of DNA double-strand break (DSB) repair (nonhomologous end-joining (NHEJ) and homologous recombination repair (HRR)) as well as in cell cycle and transcriptional regulation [1-7]. This wide range of BRCA1 activities may be attributed to the association with numerous proteins part of the BRCA1-associated genome surveillance complex (BASC) [8-10]. Many of the proteins found in the BASC have known roles in the DNA damage response (DDR) and DSB repair and appear to have a dynamic relationship with BRCA1. As an integral part of this complex, BRCA1 might serve as a scaffolding protein to help facilitate localization of repair factors and coordinate the temporal assembly and disassembly of proteins at the DSB necessary for efficient repair (for a recent review [11]). Therefore, it is no surprise that mutations in the *BRCA1* gene affect the DDR in ways that lead to ineffective or faulty DNA repair.

Most cancer-causing mutations within the *BRCA1* gene have been found in the N- and C-terminal regions of the protein [12]. The N-terminal RING domain interacts with the BARD1 protein to form a hetero-dimeric complex with E3 ubiquitin-ligase activity. In addition, the BRCA1 C-terminal (BRCT) repeats are known to bind proteins having a unique phospho-serine motif (pS-X-X-F) that are involved in DNA damage checkpoint regulation and DSB repair [13-18]. It is believed that mutations within these regions result in loss of BRCA1 tumor suppressor function leading to genomic instability and, ultimately, the onset of breast and ovarian cancers [12, 19]. A functional link between the BRCA1 RING and BRCT domains was established with the finding that BRCA1-BARD1 ubiquitinates the BRCT-interacting protein CtIP resulting in CtIP-mediated G2/M arrest and localization into DNA damage-induced foci [20]. However, the BRCT domain also binds the RAP80 ubiquitin-binding protein (through Abraxas), MERIT40, and other proteins that are not ubiquitinated such as the BRIP1 helicase, all of which are intimately associated with HRR [16-18, 21-25]. It is now clear that BRCA1 plays a major role in the organization and assembly of proteins at DSBs, and this coordination is fine-tuned by SUMO (small ubiquitin-related modifiers) and ubiquitin post-translational modifications to ensure efficient HRR [26-29].

In the present study, we show that disruption of phospho-protein binding to the BRCA1 BRCT domain leads to hyper-recombination, perhaps as a result of excessive DNA resection. We further show disrupted binding increases ubiquitination of the BASC complex which is necessary for hyper-recombination. The naturally occurring BRCA1 BRCT mutant M1775R also produced unusual looking promyelocytic leukemia nuclear bodies (PML-NBs), suggesting M1775R upsets the normal processing of the BASC. Our results suggest that binding of repair proteins to the BRCT domain, together with timely (de)ubiquitination, is vital for correct spatiotemporal processing of DSBs and coordination of HRR. Thus, disruption of proper communication between proteins binding to the BRCT domain and ubiquitination may lead to increased genomic instability due to aberrant hyper-recombination, and perhaps the onset of breast cancer.

## RESULTS

### K1702M induces hyper-recombination resulting from reduced binding to the BRCT domain

**Figure 1.**
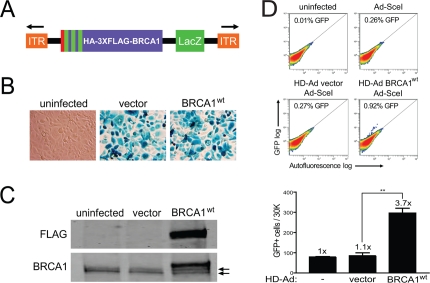
Rescue of HRR in HCC1937 cells with the expression of BRCA1 from a helper-dependent adenoviral (HD-Ad) vector. **(A)** Schematic representation of the HD-Ad vector co-expressing a combined N-terminal influenza-hemagglutinin (HA) and triple-FLAG (3XFLAG) epitope tagged BRCA1 and the β-galactosidase (LacZ) reporter (ITR, inverted terminal repeat). **(B)** LacZ staining for β-galactosidase expression after infection with HD-Ad vector control or wild-type BRCA1 (BRCA1^wt^) or with uninfected HCC1937 cells. **(C)** Western blot analysis of lysates from HCC1937 cells infected as in **B** probed with FLAG (HD-Ad BRCA1) and BRCA1 (HD-Ad BRCA1 + endogenous 5382insC BRCA1) antibodies. Arrows indicate protein bands related to endogenous 5382insC BRCA1. **(D)** HCC1937/DR-GFP cells were infected with the indicated HD-Ad vectors or left uninfected followed by infection with Ad-SceI and HRR/GFP events were analyzed by fluorescence-activated cell sorting (FACS). Error bars show SEM from three independent experiments (**, *P* < 0.01). *P* = 0.0017 when wild-type BRCA1 was compared to vector control.

To efficiently express full-length BRCA1 in HCC1937 cells, which are a model cell system for elucidating BRCA1 function and are homozygous for the *BRCA1* 5382insC mutation [30], we generated helper-dependent adenoviral (HD-Ad) vectors co-expressing BRCA1 and a LacZ reporter [31] (Figure 1A). The benefits of expressing BRCA1 from adenovirus are several-fold in that gene copy number and expression levels can be fully controlled without imposing possible cell selection and expression artifacts resulting from stable and transient expression from plasmids. To test the infectivity of HCC1937 cells, we performed β-galactosidase (LacZ) staining and found high levels of infection efficiency (Figure 1B). We also confirmed expression of full-length BRCA1 by western blot analysis and found that it migrated slightly above the endogenous, truncated BRCA1 protein (erroneous translation distal to codon 1755) produced by HCC1937 cells (Figure 1C). The level of endogenous, truncated BRCA1 to virus-expressed BRCA1 was similar (2.6-fold above endogenous), which is critical since too much BRCA1 expression could possibly offset desired reconstituted effects. These results show that HD-Ad vectors infect HCC1937 cells with high yield and express full-length BRCA1 transiently at close to physiological levels.

Having demonstrated BRCA1 expression using the HD-Ad vector system, we next determined the effect of BRCA1 on HRR in HCC1937 cells using the green fluorescent protein (GFP)-based I-*Sce*I assay [32-34]. We found that BRCA1 significantly increased HRR almost 4-fold compared to vector control infected cells, which showed repair levels similar to those of uninfected cells (Figure 1D). These results demonstrate that the DSB repair defect of HCC1937 cells can be rescued by BRCA1 expressed from HD-Ad, in line with previous reports [1, 35].

**Figure 2.**
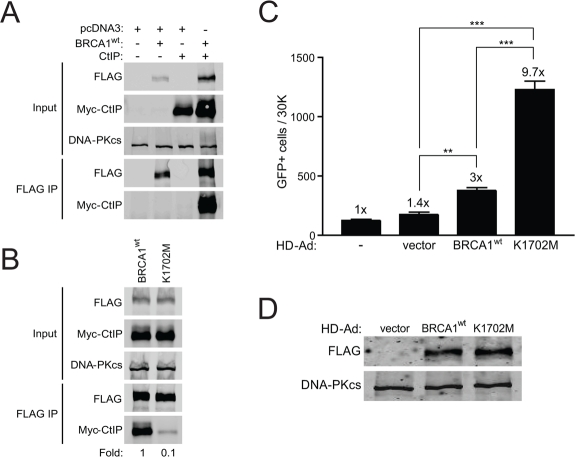
K1702M binds CtIP poorly relative to wild-type BRCA1 and results in hyper-recombination. **(A)** HEK293T cells were co-transfected with plasmids expressing Myc-CtIP, FLAG-BRCA1^wt^, or empty vector (pcDNA3) where indicated. Total cell lysates were immuno-precipitated with anti-FLAG beads and examined for CtIP binding by western blot analysis. DNA-PKcs was used as a loading control. **(B)** FLAG-BRCA1^wt^ or K1702M were co-transfected with Myc-CtIP and immuno-precipitated FLAG material was examined for CtIP binding as in **A**. **(C)** HCC1937/DR-GFP cells infected with the indicated HD-Ad vectors or left uninfected were analyzed for HRR/GFP events by FACS. Error bars show SEM from three independent experiments (**, *P* < 0.01; ***, *P* < 0.001). *P* = 0.0028 and 0.0004 when wild-type BRCA1 was compared to vector control and K1702M, respectively. *P* = 0.0001 when vector control was compared to K1702M. **(D)** Western blot analysis for FLAG-BRCA1 expression in infected HCC1937 cells with DNA-PKcs as loading control.

The K1702 residue resides within the binding pocket of the BRCA1 BRCT domain and interacts directly with the phospho-serine moiety of pS-X-X-F motif containing proteins that bind to the pocket [36]. Thus, we expected that the K1702M mutation would result in decreased binding of BRCT interacting proteins such as CtIP, which served as an indicator of the ability of this mutant to bind any phospho-protein in our study. Plasmids expressing wild-type or K1702M BRCA1 were co-transfected with a plasmid expressing CtIP into HEK293T cells followed by immuno-precipitation of FLAG-BRCA1. As expected, we found that wild-type BRCA1 bound CtIP efficiently (Figure 2A). We also noticed an increase in the abundance of BRCA1 and CtIP when co-expressed compared to either alone, suggesting that complex formation increases the stability of these two proteins. When we examined binding of CtIP to K1702M, we found at least a 10-fold reduction compared to wild-type BRCA1 (Figure 2B). These results demonstrate that the K1702M mutation disrupts the binding of CtIP*in vivo*, which is consistent with *in vitro* binding studies of peptides [36, 37]. Presumably, this disruption also extends to other phospho-proteins that normally bind the BRCA1 BRCT domain. Thus, the K1702M phenotype would be a reflection of the inability of any pS-X-X-F protein to bind BRCA1 and influence DSB repair.

We next determined the effect of K1702M on HRR. As the BRCA1 BRCT domain is known to interact with CtIP, BRIP1, and Abraxas-RAP80, which are all involved in HRR [16-18, 21, 22, 38], we expected to see a decrease in HRR with K1702M. Surprisingly, we found that K1702M significantly increased HRR >3-fold compared to wild-type BRCA1 (Figure 2C). In addition, when we examined what effect K1702M might have on NHEJ levels at a separate locus, we found that NHEJ increased 2-fold, similar to cells infected with wild-type BRCA1 virus (Supplemental Figure S1). This result suggests that the effect of K1702M on DSB repair is HRR-specific. Curiously, K1702M hyper-recombination did not increase the radiosurvival of HCC1937 cells, whereas wild-type BRCA1 did (Supplemental Figure S2). In fact, K1702M cells were more radiosensitive than wild-type BRCA1 cells, even though expression levels were similar (Figure 2D).

To further investigate the underlying cause of K1702M-mediated hyper-recombination, we determined whether this mutant had any effect on cell-cycle distribution. We found that K1702M resulted in almost a 3-fold increase of cells in the G2/M phases, and a corresponding decrease in the G1 phase, over a 48 hour time period compared to wild-type BRCA1 (Supplemental Figure S3). In addition, there were little to no differences in the cell cycle profile between vector control and wild-type BRCA1 infected cells. Because HRR occurs predominantly, if not exclusively, in late S and G2 [39, 40], these cell cycle perturbations as well as decreased radiosurvival suggest a possible defect in HRR of spontaneous and radiation-induced DSBs in cells expressing the K1702M mutant. Thus, deregulated HRR in cells with reduced binding of phospho-proteins to BRCA1, manifested as hyper-recombination in the DR–GFP assay, could be more of an impediment for cell survival than a null mutation of a HRR gene, perhaps as a result of aberrant recombination events.

### K1702M increases RAD51 nuclear staining that co-localizes with RPA

**Figure 3.**
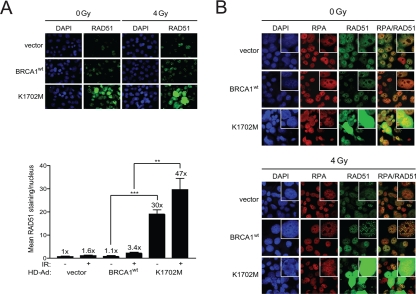
K1702M increases RAD51 foci and nuclear immuno-staining that co-localizes with RPA in irradiated HCC1937 cells. **(A)** HCC1937 cells infected with the indicated HD-Ad vectors were irradiated with 4 Gy or left unirradiated and stained for RAD51. Error bars show SEM from three independent experiments of 10 random fields with at least 100 cells per sample (**, *P* < 0.01; ***, *P* < 0.001). *P* = 0.0007 and 0.0053 when wild-type BRCA1 and K1702M unirradiated and irradiated cells were compared, respectively. The percentage of cells having bright RAD51 staining was <1% for wild-type BRCA1 and 4-7% for K1702M. **(B)** HCC1937 cells immuno-stained for RAD51 in **A** were re-stained for RPA.

To determine whether the hyper-recombination observed with K1702M in the site-specific DR-GFP assay reflected an overall increase in HRR in the cell, we next examined RAD51 foci formation in irradiated cells. RAD51 is uniquely utilized in HRR and the appearance of RAD51 foci indicate repair centers engaged in HRR [41]. In support of the results seen with the DR-GFP assay, without any irradiation K1702M expressing cells showed significant ~30-fold higher levels of RAD51 nuclear staining than cells expressing wild-type BRCA1 (Figure 3A). However, only an ~1.5-fold further increase was observed with K1702M cells after irradiation. This result suggests that spontaneous DSBs, e.g., lesions generated during replication, could account for a large proportion of the increased RAD51 nuclear staining in K1702M cells. Interestingly, western blot analysis showed no increase in the overall levels of RAD51 in lysates from K1702M compared to those from cells expressing wild-type BRCA1 (Supplemental Figure S4). This result suggests it is the localization of RAD51 at defined DNA sites that is enhanced rather than the overall level of RAD51 expression when K1702M is expressed. Altogether, these results suggest that K1702M promotes the generation of extensive RAD51 filaments regardless of exogenous DNA damage, a finding that is in line with the increased levels of HRR seen with the DR-GFP assay.

When a DSB is processed for repair by HRR, the 5' DNA ends are resected to provide 100-200 nucleotides or more of 3' DNA overhangs suitable for strand-invasion, Holliday junction formation, and the completion of repair [42]. To determine whether K1702M expression might increase ssDNA formation, we examined whether RPA co-localized with RAD51 in these cells. RPA binds to ssDNA and stabilizes Holliday junction intermediates. Indeed, RPA was found to co-localize with RAD51, which increased after irradiation in both BRCA1 wild-type and K1702M infected cells (Figure 3B). However, the tremendous increase in nuclear RAD51 in a sub-population of K1702M cells overwhelmed the RPA staining making it impossible to determine whether co-localization occurred or not. These results support the notion that K1702M promotes extensive ssDNA formation that results in aberrant DNA strand-exchanges.

### K1702M increases ubiquitination of the BASC necessary for hyper-recombination

**Figure 4.**
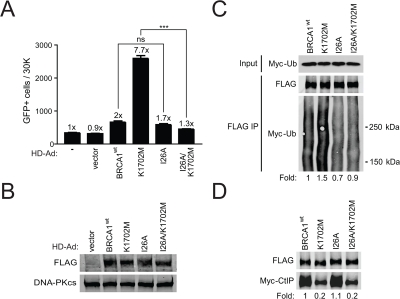
K1702M increases ubiquitination of the BASC which is required for hyper-recombination. **(A)** HCC1937/DR-GFP cells infected with the indicated HD-Ad vectors were analyzed for HRR/GFP events by FACS. Error bars show SEM from three independent experiments (***, *P* < 0.001; ns, not significant). *P* < 0.0001 when K1702M was compared to I26A/K1702M. *P* = 0.1780 when wild-type BRCA1 was compared to I26A. **(B)** Western blot analysis for FLAG-BRCA1 expression in infected HCC1937 cells with DNA-PKcs as loading control. **(C)** HEK293T cells were co-transfected with Myc-ubiquitin plasmid and the indicated FLAG-BRCA1 plasmids. Immuno-precipitated FLAG material was examined for ubiquitin by western blot analysis. The smear in each of the lanes represents the many proteins in the BASC that are ubiquitinated. **(D)** The indicated FLAG-BRCA1 plasmids were co-transfected with Myc-CtIP plasmid and immuno-precipitated FLAG material was examined for CtIP binding by western blot analysis.

As BRCA1 is known to ubiquitinate CtIP [20], we determined the effect of decreased CtIP binding on ubiquitination of the BASC and the functional link between BASC ubiquitination and HRR using an I26A/K1702M double mutant. The BRCA1 I26A ubiquitin-ligase mutant is known to prevent the interaction of BRCA1 with the E2 ubiquitin-conjugase while still binding BARD1 [43]. We found that the double mutant significantly diminished hyper-recombination compared to the K1702M single mutant when BRCA1 expression levels were similar (Figure 4A, B). In addition, we did not observe a significant difference in HRR between wild-type BRCA1 and the I26A single mutant in agreement with a previous report [44]. To determine whether diminished hyper-recombination correlated with decreased BASC ubiquitination, plasmids expressing different BRCA1 forms were co-transfected with a plasmid expressing Myc-tagged ubiquitin into HEK293T cells. We found that K1702M increased ubiquitination of the BASC ~1.5-fold compared to wild-type BRCA1 (Figure 4C). As expected, I26A showed decreased (~0.7-fold) ubiquitination of the BASC but retained the ability to bind CtIP at levels similar to wild-type BRCA1, whereas the double mutant displayed a similar defect in CtIP binding as the K1702M single mutant (Figure 4C, D). Our results show that hyper-recombination requires a ubiquitination-dependent interaction between the BRCA1 RING and BRCT domains, a process that is enhanced when phospho-proteins are unable to bind to the BRCT domain. Altogether, these results suggest that BASC ubiquitination correlates with hyper-recombination and is dominant over the BRCT-mediated processes caused by defective phospho-protein binding.

### M1775R increases RAD51 and RPA pan-nuclear staining in part co-localizing with ssDNA but not with atypical PML nuclear bodies

**Figure 5.**
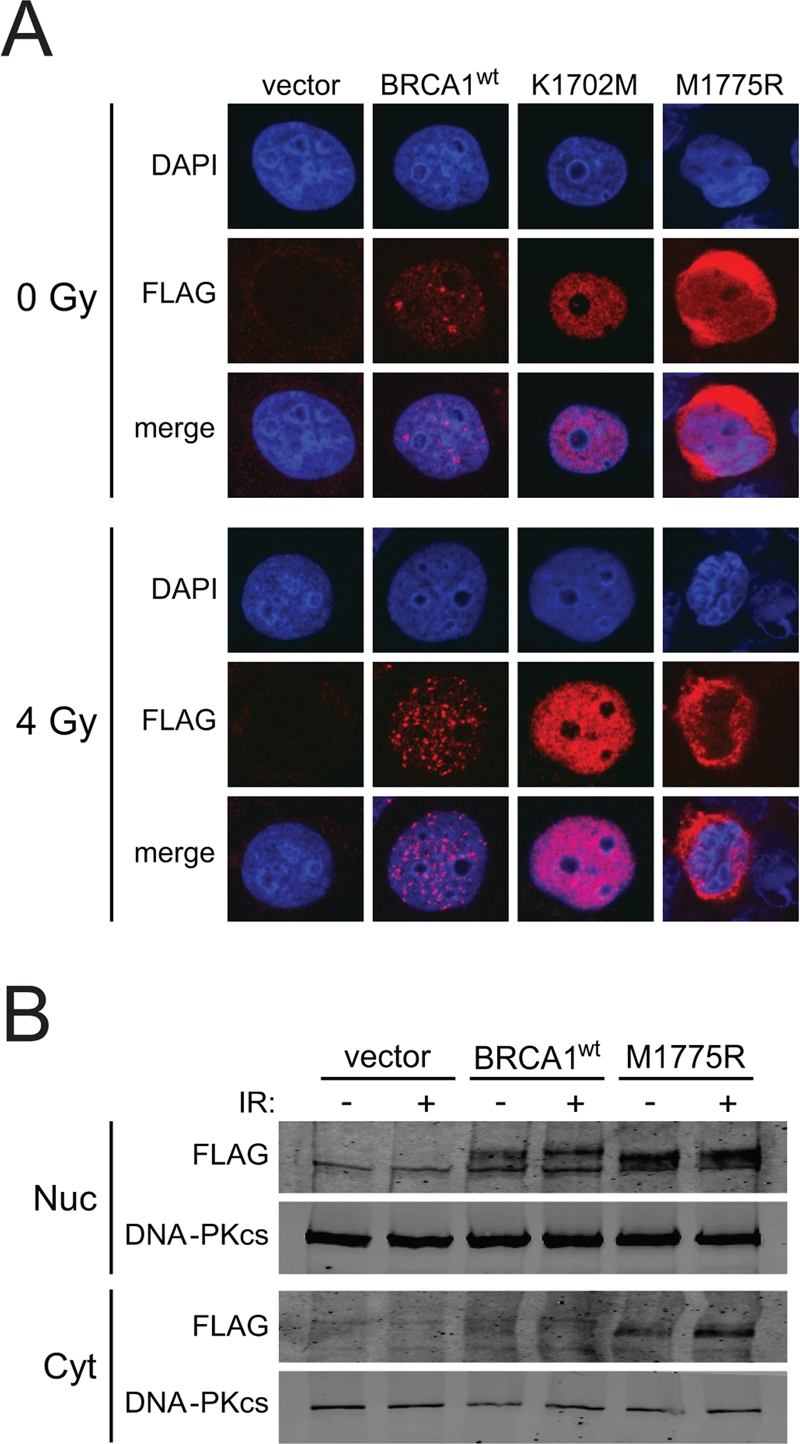
BRCA1 BRCT mutants expressed from HD-Ad vectors enter the nucleus of HCC1937 cells. **(A)** HCC1937 cells infected with the indicated HD-Ad vectors were examined for BRCA1 nuclear localization by FLAG immuno-staining after 4 Gy irradiation. Representative nuclei are shown. **(B)** HCC1937 cells infected with the indicated HD-Ad vectors were treated as in **A** and fractionated to examine FLAG-BRCA1 localization by western blot analysis. DNA-PKcs was used as a marker for determining the yield and efficiency into nuclear and cytoplasmic fractions.

M1775R is a recurring deleterious BRCA1 mutation and one of the first reported missense mutations of BRCA1 [45-47]. The M1775R mutation prevents phospho-protein binding by sterically clashing with the phenylalanine ring of the pS-X-X-F motif as opposed to K1702M, which disrupts the electrostatic interaction of K1702 with the phospho-serine of the motif [36, 37, 48]. Previous studies have suggested that M1775R is impaired in nuclear localization and foci formation [36, 49, 50]. Therefore, we first examined whether BRCT mutants expressed from the HD-Ad vectors were able to enter the nucleus of HCC1937 cells. K1702M showed enhanced nuclear localization compared to wild-type BRCA1, regardless of irradiation (Figure 5A). In addition, M1775R also entered the nucleus but seemed to remain around the nuclear membrane. To substantiate this result, we conducted a cell fractionation experiment followed by western blotting and found that most of M1775R was indeed located in the nuclear fraction with some protein remaining in the cytoplasm, while there was little to no detectable wild-type BRCA1 in the cytoplasm regardless of whether cells were irradiated or not (Figure 5B). In the nuclear fraction, wild-type BRCA1 had slower electrophoretic mobility than M1775R, suggesting that the wild-type protein was more heavily modified than the mutant. These results suggest that while there may be some impairment to M1775R nuclear localization as previously reported [36, 49, 50], significant levels of BRCA1 BRCT mutant proteins were present in the nucleus following their expression from HD-Ad vectors, which would be necessary to influence HRR.

**Figure 6.**
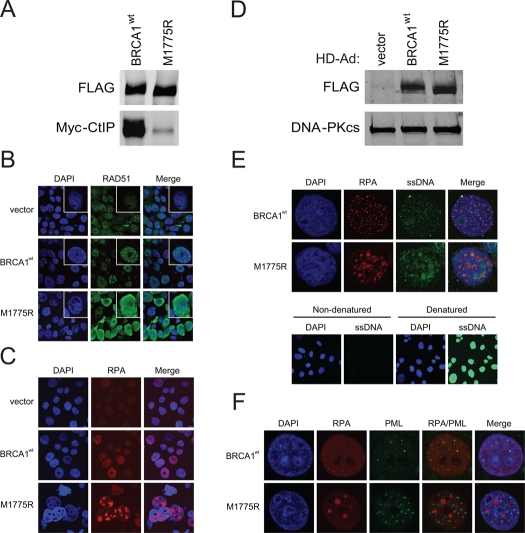
M1775R is defective in CtIP binding and increases RAD51 and RPA pan-nuclear immuno-staining that in part co-localizes with elevated ssDNA formation but not atypical PML-NBs. **(A)** HEK293T cells were co-transfected with Myc-CtIP plasmid and the indicated FLAG-BRCA1 plasmids. Immuno-precipitated FLAG material was examined for CtIP binding by western blot analysis. **(B)** RAD51 staining of unirradiated HCC1937 cells infected with the indicated HD-Ad vectors. **(C)** RPA staining of representative HCC1937 cells treated as in **B**. The percentage of cells having large, clustered RPA foci was <1% for wild-type BRCA1 and 7-10% for M1775R. **(D)** Western blot analysis for FLAG-BRCA1 expression in infected HCC1937 cells with DNA-PKcs as loading control. **(E)** RPA and ssDNA co-staining of representative HCC1937 cell nuclei treated as in **B** and **C**. Cells labeled in parallel with BrdU were denatured to determine the extent of incorporation. **(F)** RPA and PML co-staining of representative HCC1937 cell nuclei treated as in **B**, **C**, and **E**. The percentage of cells having thread-like PML-NBs was <1% for wild-type BRCA1 and >50% for M1775R.

M1775R also demonstrated a much reduced ability to bind CtIP and produced elevated levels of RAD51 and RPA nuclear staining compared to wild-type BRCA1 when BRCA1 expression levels were similar, all of which were not dependent on exogenous DNA damage (Figure 6A-D). In fact, M1775R induced the formation of what appeared to be large, clustered RPA foci. To determine whether the increased nuclear RAD51 and RPA staining was indeed the result of increased ssDNA engaged in recombination, we examined the extent of ssDNA formation by an assay that utilizes BrdU incorporation into DNA [41]. Indeed, M1775R cells showed elevated levels of ssDNA that to some extent co-localized with the large clusters of RPA foci compared to the much smaller co-localized foci seen in wild-type BRCA1 cells (Figure 6E). Furthermore, in cells expressing wild-type BRCA1, RPA was juxtaposed with promyelocytic leukemia (PML) nuclear bodies (NBs) whereas cells expressing M1775R showed highly unusual, thread-like PML-NBs that did not co-localize with RPA (Figure 6F). PML-NBs are believed to be nuclear compartments for post-translational processing such as (de)SUMOylation and (de)ubiquitination and the (de)assembly of protein complexes critical for transcription and DNA repair [51, 52]. Recently, it was shown that SUMOylation is important for efficient HRR [28, 29]. Thus, the appearance of atypical, thread-like PML-NBs in M1775R but not wild-type BRCA1 expressing cells suggests that M1775R promotes the generation of these abnormal PML-NBs. All combined, the naturally occurring M1775R mutant generated a similar, if not more dramatic, phenotype compared to K1702M in terms of RAD51 and RPA nuclear staining, but also showed evidence of extensively resected DNA and unusual looking PML-NBs, perhaps indicative of stalled repair.

### M1775R increases nuclear RAD51 and RPA staining in human breast cancer tissue

**Figure 7.**
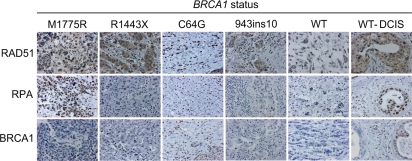
RAD51 and RPA levels are elevated in human breast cancer tissue with a BRCA1 M1775R mutation. Human breast cancer tissue sections with the indicated *BRCA1* germline mutations were immuno-stained for RAD51, RPA, and BRCA1 (WT, breast tumor with wild-type *BRCA1* status; DCIS, ductal carcinoma *in situ*). Shown are representative malignant regions scored in yellow highlight in Supplemental Table S1.

We next determined whether our *in vitro* results would translate into effects seen in human breast cancer tissue. Sections from patients with various BRCA1 mutations, including M1775R and C64G (reported to have a similar defect in ubiquitin-ligase activity as the synthetic I26A mutation [43]), and patients with normal BRCA1 breast tumors were examined. Consistent with our *in vitro* data, the M1775R tissue section showed increased RAD51 and RPA staining in nuclear malignant regions (Figure 7; Supplemental Table S1). In fact, the RPA levels in M1775R were the highest of all the sections and similar to those of a ductal carcinoma *in situ* (DCIS) section (WT-DCIS). However, the M1775R section did not show any detectable BRCA1 staining, similar to the R1443X and 943ins10 sections. This result suggests that M1775R is down-regulated, perhaps due to toxicity and/or instability *in vivo* as reported *in vitro* [53]. This finding implies that considering the relatively low expression, M1775R results in very high relative levels of RAD51 and RPA if these are dependent on BRCA1 compared to those seen with either the R1443X or 943ins10 sections that also had low BRCA1 expression. On the other hand C64G, which is known to increase the stability of BRCA1 by oligomerization [54], showed high levels of RAD51, RPA, and BRCA1. Thus, the level of BRCA1 expression should be taken into consideration when assessing the impact of BRCA1 on HRR *in vivo*. Based on the high expression of RAD51 and RPA relative to low M1775R expression, these results suggest that M1775R may promote excessive HRR *in vivo* as has been suggested to occur in some breast cancers with low BRCA1 levels [55]. However, more tissues with BRCT mutations need to be screened before such an assertion could be made significant. We also observed elevated levels of RAD51 and RPA staining in a DCIS tissue section with wild-type BRCA1 that showed increased BRCA1 expression relative to malignant tissue. DCIS is commonly associated with non-invasive, pre-malignant breast cancer. Thus, the data presented here could perhaps have clinical implications in that RAD51 and RPA might be useful diagnostic markers for pre-malignant (DCIS) breast cancers and, in particular, for screening breast cancers for mutations in the BRCA1 BRCT domain that result in hyper-recombination.

## DISCUSSION

In this study, we examined the ability of synthetic and natural BRCA1 BRCT mutants to modulate DSB repair. As a surrogate for determining protein binding to the BRCT domain, we used CtIP which is known to bind BRCA1 efficiently when phosphorylated at S327 [56]. However, it is presumed that these BRCT mutants would not bind any protein with a pS-X-X-F motif, also including Abraxas-RAP80, BRIP1, and MERIT40 [11]. As expected, we found that both the K1702M and M1775R mutants were severely impaired in CtIP binding. Surprisingly, we found that HCC1937 cells expressing these BRCT mutants displayed hyper-recombination by the DR-GFP assay and increased RAD51-RPA-ssDNA nuclear staining, indicative of elevated levels of recombination intermediates. There is precedence for BRCA1-associated hyper-recombination as several recent reports suggest that the disruption or depletion of specific BRCA1-interacting proteins results in hyper-recombination [57-59]. Our results further show that cells expressing BRCT mutants accumulate in G2 and require ubiquitination for hyper-recombination. BRIP1 is believed to mainly function in S and not be dependent on ubiquitination [60], whereas Abraxas-RAP80 and CtIP function primarily in G2 and depend on ubiquitination [17, 20]. Of the three better characterized BRCT binding partners (Abraxas-RAP80, BRIP1, and CtIP), we favor the disruption of Abraxas-RAP80 and CtIP binding to BRCA1 as basis for the hyper-recombination we observe in our study.

A similar stimulation of HRR to what we see with BRCT mutants was recently demonstrated when either Abraxas, RAP80, or BRCC36 was depleted [58, 59]. It was proposed that the role of the BRCA1-Abraxas-RAP80-BRCC36 complex is to limit DSB resection. Thus, disruption of this complex by certain BRCA1 BRCT mutations may lead to further resection mediated by CtIP or other nucleases. CtIP has important roles in both HRR and NHEJ, and based on its similarity to the yeast Sae2 protein, which harbors nuclease activity toward a variety of defined repair substrates *in vitro*, CtIP acts as both an adaptor and nuclease in mammalian DSB processing [38, 61]. Silencing CtIP or expression of a S327A mutant unable to bind BRCA1 impairs HRR [38]. BRCA1 also associates with the MRE11-RAD50-NBS1 (MRN) complex [62], and BRCA1-CtIP-NBS1 form a complex in a cell-cycle dependent manner [23]. In addition, in human cells Exo1 works together with CtIP and MRN to resect the DSB [63]. Thus, it might be the improper function of the BRCA1-Abraxas-RAP80 complex, combined with untamed CtIP, MRN, and/or Exo1 resection that leads to the generation of more extensive ssDNA at the DSB in our study.

It is important to point out that the increased HRR we see with the DR–GFP assay is likely a reflection of an increased rate by which Holliday junctions are formed and remain present rather than an increased number of DSBs becoming available for processing. The single I–*Sce*I target site is likely cleaved to the same extent regardless of BRCA1 status, and the K1702M mutation did not appear to change the fraction of breaks being channeled into NHEJ in agreement with one study [58], but in disagreement with another [59]. The reason for these dissimilarities is unclear but could be explained by differences in experimental system or cell cycle effects. In our study, when K1702M was expressed we noticed an accumulation of cells in G2 where HRR is particularly active. Thus, excessive DNA resection could accelerate progression to the later stages of HRR which may be the main contributing factor to the faster accumulation of GFP+ cells we see with the K1702M mutant.

We also found that K1702M increased ubiquitination of the BASC, and that ubiquitination was necessary for hyper-recombination but did not affect the ability of wild-type BRCA1 to stimulate HRR. It was recently shown that the BRCA1 I26A ubiquitin-ligase mutant did not affect HRR and mitomycin C survival of mouse embryonic stem cells [44], which is consistent with our results. Thus, ubiquitination is necessary for hyper-recombination in the context of altered BRCT interactions to compensate for the loss of normal repair activity associated with BRCA1 complexes. It is tempting to speculate that BRCC36, part of the Abraxas-RAP80 complex and a putative deubiquitinase [24], perhaps cannot deubiquitinate the BASC, resulting in an accumulation of ssDNA intermediates and RPA-RAD51-DNA complexes unable to turn over. Therefore, increased DSB resection could be the result of a failure of BRCT mutants to support the progression of repair past resection due to the inability of BRCC36, or other deubiquitinase, to process late-stage repair complexes. Ubiquitination of chromatin necessary for the binding of BRCA1 to DSBs through Abraxas-RAP80 is required early in the DDR [16-18]. Therefore, we favor the idea that BRCT mutants are able to overcome this early blockade. Instead, it seems it is a later stage of the repair process following DNA resection, which is also critically ubiquitination-dependent, that primarily contributes to hyper-recombination.

Promyelocytic leukemia nuclear bodies (PML-NBs) are important for the assembly and disassembly of protein complexes, including those with RAD51, RPA, and BRCA1 necessary for HRR (for a recent review [51]). The finding that M1775R promoted clustered RPA foci that in part co-localized with ssDNA but not with the atypical PML-NBs is interesting. If (de)SUMOylation-(de)ubiquitination is necessary for the spatiotemporal processing of HRR complexes that might occur in or in close proximity to PML-NBs [51,52], this suggests that M1775R is upsetting some step after Holliday junction formation resulting in the creation of these unusual PML-NBs. Atypical PML-NBs may be a manifestation of altered (de)ubiquitination of the BASC as discussed above. What is presently not clear is whether the cells with clustered RPA foci and atypical PML-NBs represent a temporary accumulation of intermediates in a continuum of events leading to hyper-recombination or whether these cells are irreversibly ‘stuck’ in the repair process. Thus, certain BRCT mutants might upset the spatiotemporal ‘handing-over’ processes controlled by (de)ubiquitination which is seen as an accumulation of late repair intermediates and hyper-recombination.

To date, little is known about how pathogenic BRCA1 mutations that disrupt BRCT phospho-protein binding affect DSB repair and the pathophysiology of breast cancer [55, 64]. The results from our limited analysis of breast cancer tissues, including one from a patient with the M1775R mutation, are in line with our *in vitro* findings and the notion that RAD51 and RPA foci levels are elevated in cells that express M1775R. It was previously reported that some BRCA1-deficient breast tumors have enhanced HRR [55]. However, the germ-line *BRCA1* statuses of these samples were not identified. The results presented here help explain how BRCA1 BRCT mutations might contribute to breast cancer through a mechanism involving increased, and most likely aberrant, HRR rather than through the loss of BRCA1 function and reduced HRR. In fact, our radiosurvival experiment suggests that K1702M radiosensitizes cells rather than just fails to rescue radiosensitivity. Similar conclusions were also made to explain genomic instability and increased radiosensitivity of cells that were depleted of RAP80, Abraxas, or BRCC36 [58, 59]. Our results are complementary to these recent findings and recapitulate hyper-recombination with specific BRCT mutants (one of them naturally occurring) unable to bind critical proteins, and link genomic instability with ubiquitination-dependent processing that occurs late in HRR. Most importantly, we have demonstrated that the correct temporal and physical interactions between BRCA1 and these proteins are critical for maintaining normal HRR and preserving genomic stability. Our results have implications for the development of potential inhibitors disrupting BRCT interactions. Such inhibitors might actually stimulate HRR and lead to cell-cycle arrest with relatively little impact on the radiosurvival of tumor cells with normal BRCA1. On the other hand, the finding that ubiquitination dominates over the BRCT-mediated effects resulting in hyper-recombination suggests that targeted inhibition of BRCA1 ubiquitin-ligase activity or enhancing deubiquitination could be more effective for interfering with BRCA1-mediated DSB repair in BRCT mutant cells. Thus, caution should be exercised when targeting BRCA1 for therapeutic intervention.

## METHODS

### Cell culture

HCC1937 cells were cultured at 37°C in RPMI-1640 (Cellgro), and HEK293-116C and HEK293T cells in DMEM (Gibco) medium supplemented with 10% FBS and antibiotics.

### Plasmids

QuikChange site-directed mutagenesis (Stratagene) was used to create mutations from wild-type (wt) BRCA1 plasmid pcDNA3(*BssH*II)-HA-3XFLAG-BRCA1. The K1702M mutation was created using primers 5'-GTGAACGGACACTGATGTATTTT CTAGGAATTG-3' and 5'-CAATTCCTAGAAAATAC ATCAGTGTCCGTTCAC-3'. The I26A mutation was created using primers 5'-CTTAGAGTGTCCCGCCTG TCTGGAGTTG-3' and 5'-CAACTCCAGACAGGCGG GACACTCTAAG-3'. The M1775R mutation was created using primers 5'-TATGGGCCCTTCACCAAC AGACCCACAGATCAACTGG-3' and 5'-CCAG TTGATCTGTGGGTCTGTTGGTGAAGGGCCCATA-3'. Additional plasmids used were pcDNA3-5XMyc-CtIP and pCMV-Myc-ubiquitin [65, 66].

### Generation of HD-Ad vectors

Plasmids pcDNA3(*BssH*II)-HA-3XFLAG-BRCA1 wt, K1702M, I26A, I26A/K1702M, and M1775R were digested with *BssH*II and the HA-3XFLAG-BRCA1 fragments ligated into the *Asc*I site of the helper-dependent adenovirus (HD-Ad) plasmid pΔ28E4LacZ [31]. The pΔ28E4LacZ-HA-3XFLAG-BRCA1 plasmids were digested with *Pme*I to release the viral DNA from the plasmid backbone. The viral DNA was packaged into adenovirus and amplified by transfecting HEK293-116C cells using Superfect (Qiagen) as described [31].

### Antibodies

Antibodies used were FLAG (M2; Sigma), BRCA1 (Ab-1; Calbiochem), Myc (9B11; Cell Signaling), RAD51 (Ab-1; Calbiochem), DNA-PKcs (4F10C5; Biosource), RPA/p32 (Ab-1; NeoMarkers), BrdU (BU1/75 (ICR1); Abcam), PML (H-238; Santa Cruz Biotechnology), and Actin (I-19; Santa Cruz Biotechnology).

### Western blotting and immuno-precipitation

All cellular material was prepared 48 h after plasmid transfection or HD-Ad infection and proteins were separated by SDS-PAGE for western blot analysis. Total cell lysates were prepared in RIPA buffer with protease and phosphatase inhibitors (Sigma) and PMSF. For immuno-precipitation experiments, recombinant HA-3XFLAG-BRCA1 protein from HEK293T cells transfected with pcDNA3(*BssH*II)-HA-3XFLAG-BRCA1 plasmids using polyethylenimine (Polysciences, Inc.) was immuno-precipitated with anti-FLAG M2 beads (Sigma) in 0.5X RIPA buffer/0.5X PBS with inhibitors. For cell fractionation, cell pellets were treated with a 0.5% Triton X-100 lysis buffer to release cytoplasmic proteins followed by centrifugation (2,000 rpm) and nuclei were washed in lysis buffer and PBS followed by centrifugation. Nuclei were then incubated in Dignam nuclear buffer for 30 min and centrifuged for 30 min at 10,000 rpm. Volumes were adjusted to give equal portions of cytoplasmic and nuclear fractions. Protein was imaged and quantified using the Odyssey Infrared Imaging System and application software version 3.0 (Li-Cor Biosciences).

### DNA repair assay

The HRR-GFP repair system has been described previously [33, 34]. HCC1937 cells were stably transfected with the DR-GFP repair plasmid [32], and selected in puromycin. HCC1937/DR-GFP cells at ~90% confluency were serum-starved and infected with HD-Ad vectors. Forty-eight hours after HD-Ad infection, cells were infected with an adenovirus expressing the endonuclease I-*Sce*I (Ad-SceI) and repair events determined at either 48 or 72 h after Ad-SceI infection by GFP fluorescence-activated cell sorting (FACS) as described [33, 34].

### Immunocytochemistry

HCC1937 cells were seeded onto 4-well chamber slides and infected with HD-Ad vectors. Forty-eight hours after infection, cells were irradiated with 4 Gy using a Gammacell 40 Exactor (MDS Nordion) or left unirradiated, fixed with 3% paraformaldehyde, permeablized with 0.5% Triton X-100, immuno-stained, and counterstained with DAPI. For RAD51 and RPA staining, cells were fixed 8 h after irradiation. For RAD51 quantification, cells were imaged using the Applied Imaging Ariol (Genetix) at 40x magnification and images were quantified using CellProfiler cell image analysis software (Broad Institute) (http://www.cellprofiler.org/). Other experiments were imaged using a Zeiss LSM 510 META confocal laser scanning microscope at 63x magnification. For BRCA1 nuclear-cytoplasmic localization studies, cells were fixed and stained with FLAG antibody 4 h after irradiation. For ssDNA detection, cells were labeled with 30 μM BrdU 48 h prior to infection. Cells were then pre-extracted in buffer containing 100 mM NaCl, 1 mM EDTA, 3 mM MgCl_2_, 300 mM sucrose, and 0.5% Triton X-100 prior to fixation, and permeabilized with 0.5% Triton X-100 to detect the yield of BrdU incorporation and ssDNA. Cells were immuno-stained with anti-BrdU antibody [41], and imaged using a Leica TCS-SP2 AOBS confocal laser scanning microscope at 63x magnification. Parallel cultures were checked for total BrdU incorporation by also including a 2N HCl step followed by neutralization with 0.1 M Borax as recommended by the manufacturer.

### Selection, randomization, and blinding of human breast cancer tissues

Patients with constitutional *BRCA1* mutations were identified among all patients seen through the Clinical Genetics Service at Virginia Commonwealth University Health System. Since clinical *BRCA1* testing is exclusively available through Myriad Genetics Laboratories, Inc., Myriad provided the Clinical Genetics Service with a list of patients who were tested for *BRCA1* mutations up until September 2009. Medical records for patients with deleterious mutations were accessed by the genetic counselor in the clinical department to verify results and identify the specific mutations for these individuals. Technical specifications for *BRCA1* testing through Myriad is available (http://www.myriadtests.com/provider/doc/BRACAnalysis-Technical-Specifications.pdf). Clinic notes were also queried and one patient with a *BRCA1* M1775R mutation was identified whose testing was performed through Fox Chase Cancer Center as part of a research study. Medical record numbers of all patients (without mutation status) were sent to the study pathologist to identify patients for whom breast cancer tissue was available. The genetic counselor then assigned a study identification number to each patient with available tissue. From this list the genetic counselor selected all patients with deleterious mutations and available tissue (*n* = 4) along with two randomly selected patients among those without identified *BRCA1* mutations. The medical record numbers were then sent to the pathologist. Results were sent back to the genetic counselor at which time the data were stripped of their medical record numbers and identified only with the study identification number. In this way all investigators except the genetic counselor were blinded to the mutation status during tissue analysis and only the clinical pathologist and genetic counselor had access to patient identifiers. All procedures were approved by the VCU IRB (protocol #HM12581).

### Immunohistochemistry of human breast cancer tissues

Sectioning of tissue and antigen retrieval was performed by Anatomic Pathology Research Services, Department of Pathology, Virginia Commonwealth University. Briefly, slides were each de-paraffinized through 3 changes of xylene for 5 min and rehydrated through 2 changes of 100% reagent ethyl alcohol for 3 min, 1 change of 95% for 3 min, and 1 change of 80% for 3 min. Endogenous peroxidase activity was blocked by incubation in 0.3% hydrogen peroxide at room temperature for 5 min. Antigen retrieval was performed using Heat Induced Epitope Retrieval (HIER) consisting of a 20 min incubation in Target Retrieval Solution, pH 6 (Dako Corporation) and a 20 min cooling period. Sections were blocked with 10% normal goat serum in PBS/0.01% Triton X-100 for 1 h at room temperature. Primary antibodies RAD51 (1:1000), RPA/p32 (1:500), and BRCA1 (1:200) in Antibody Diluent with Background Reducing Agents (Dako Corporation) were added to sections and incubated overnight at 4 °C. After washing with PBS/0.01% Triton X-100, sections were incubated with Envision+^TM^ Dual Link (Dako Corporation) conjugated to HRP for 1 h at room temperature, washed again, and exposed to DAB+ (diaminobenzidine) (Dako Corporation) briefly while monitoring for the development of brown staining. Slides were then rinsed in water for several min before counterstaining with Gills III Hematoxylin (Poly Scientific). Sections were dehydrated with sequential washes in 70%, 95%, and 100% ethanol and finally incubated in Histoclear. Coverslips were then mounted over the sections with Permount (Sigma). Images of representative areas of the tumors were taken using a Nikon ACT-1 camera at 400x magnification.

### Statistics

Unpaired two-tailed t-tests were performed on data sets using GraphPad Prism 3.0 (GraphPad Software, Inc.).

## SUPPLEMENTARY FIGURE

Supplemental Figure S1. K1702M does not affect NHEJ. HCC1937/NHEJ-DsRed cells were infected with the indicated HD-Ad vectors and NHEJ levels were determined by genomic qPCR. Graph depicts relative NHEJ levels normalized to β-actin.

Supplemental Figure S2. K1702M increases the radiosensitivity of HCC1937 cells. HCC1937 cells infected with the indicated HD-Ad vectors or left uninfected were irradiated with either 4 or 8 Gy and counted by FACS after the addition of Trypan Blue. Error bars show SEM from three independent experiments (**, *P* < 0.01). *P* = 0.0022 and 0.0047 when vector control was compared to wild-type BRCA1 and K1702M at 8 Gy, respectively.

Supplemental Figure S3. K1702M arrests HCC1937 cells in S and G2. HCC1937 cells infected with the indicated HD-Ad vectors were analyzed for cell-cycle distribution by propidium iodide staining and FACS.

Supplemental Figure S4. K1702M does not increase RAD51 expression levels. Western blot analysis of RAD51 from lysates of HCC1937 cells 48 h after infection with the indicated HD-Ad vectors. Actin was used as a loading control.

## SUPPLEMENTAL TABLE

Supplemental Table S1. Pathologist's analysis of human breast cancer tissue sections.
