# Supplementary material for: Mutations in the BRCT binding site of BRCA1 result in hyper-recombination
Source: Aging (Albany NY). 2011 May 8;3(5):515–32. doi: 10.18632/aging.100325 (PMC3156602; doi:10.18632/aging.100325)
Supplement: Supplemental Figure S1. — HCC1937/NHEJ-DsRed cells were infected with the indicated HD-Ad vectors and NHEJ levels were determined by genomic qPCR. Graph depicts relative NHEJ levels normalized to β-actin. [file aging-03-515-s001.pdf]

SUPPLEMENTAL FIGURES

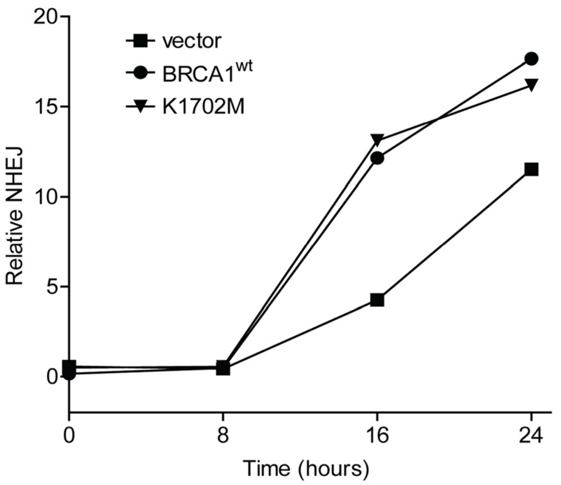

Supplemental Figure S1. K1702M does not affect NHEJ. HCC1937/NHEJ-DsRed cells were infected with the indicated HD-Ad vectors and NHEJ levels were determined by genomic qPCR. Graph depicts relative NHEJ levels normalized to β-actin.

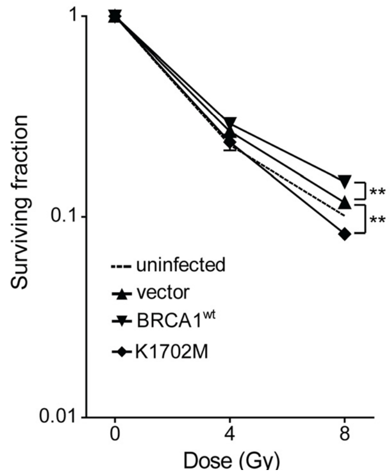

Supplemental Figure S2. K1702M increases the radiosensitivity of HCC1937 cells. HCC1937 cells infected with the indicated HD-Ad vectors or left uninfected were irradiated with either 4 or 8 Gy and counted by FACS after the addition of Trypan Blue. Error bars show SEM from three independent experiments (**, *P* < 0.01). *P* = 0.0022 and 0.0047 when vector control was compared to wild-type BRCA1 and K1702M at 8 Gy, respectively.

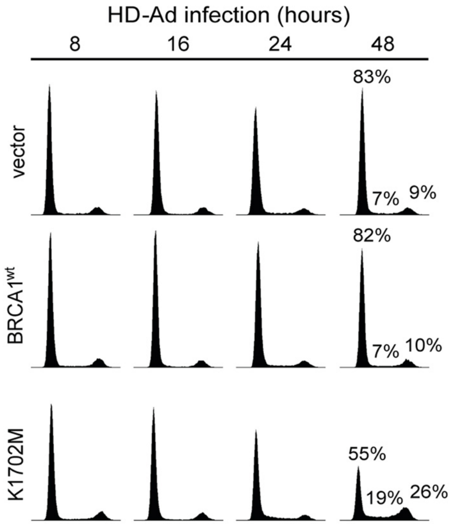

Supplemental Figure S3. K1702M arrests HCC1937 cells in S and G2. HCC1937 cells infected with the indicated HD-Ad vectors were analyzed for cell-cycle distribution by propidium iodide staining and FACS.

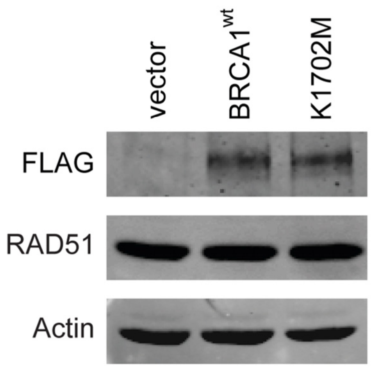

Supplemental Figure S4. K1702M does not increase RAD51 expression levels. Western blot analysis of RAD51 from lysates of HCC1937 cells 48 h after infection with the indicated HD-Ad vectors. Actin was used as a loading control.

SUPPLEMENTAL TABLE

Supplemental Table S1. Pathologist's analysis of human breast cancer tissue sections.

RAD51

ID	Germline *BRCA1* status	Nuclear benign	Nuclear malignant	Cytoplasm benign	Cytoplasm malignant	Comments
49	R1443X	2+	1+	2-3+	2+	Previous biopsy related changes and fat necrosis excluded
35	M1775R	2+	2+	1+	2+	Macrophages and fat necrosis excluded (previous biopsy site)
15	negative	NA	2+	NA	2+	Only DCIS, no benign and no invasive carcinoma
5	negative	1+	1+	2+	1+	Focal sclerosing adenosine; edge effect avoided
23	C64G	NA	2-3+	NA	2-3+	No benign breast in section
57	943ins10	1+	1+	1-2+	1-2+	Edge effect excluded

RPA

ID	Germline *BRCA1* status	Nuclear benign	Nuclear malignant	Cytoplasm benign	Cytoplasm malignant	Comments
49	R1443X	1+	2+	0	0	Previous biopsy related changes and fat necrosis excluded
35	M1775R	2+	3+	0	0	Macrophages and fat necrosis excluded (previous biopsy site)
15	negative	2+	3+	0	0	Only DCIS, no benign and no invasive carcinoma
5	negative	0	1+	0	0	Focal sclerosing adenosine; edge effect avoided
23	C64G	NA	2+	NA	0	No benign breast in section
57	943ins10	0	1+ <5%	0	0	Edge effect excluded

BRCA1

ID	Germline *BRCA1* status	Nuclear benign	Nuclear malignant	Cytoplasm benign	Cytoplasm malignant	Comments
49	R1443X	1+ Focal 1%	0	2+ Focal 1%	0	Previous biopsy related changes and fat necrosis excluded
35	M1775R	0	0	0	0	Macrophages and fat necrosis excluded (previous biopsy site)
15	negative	3+ <5%	3+ <5%	1+ <5%	1+ <5%	Only DCIS, no benign and no invasive carcinoma
5	negative	0	1+ 1%	0	1+ 1%	Focal sclerosing adenosine; edge effect avoided
23	C64G	NA	2+	NA	1+	No benign breast in section
57	943ins10	0	0	0	0	Edge effect excluded

Breast cancer tissue sections immuno-stained in Figure 7 were scored by a pathologist blinded to the study. Nuclear and cytoplasmic immuno-reactivity of the tumor cells was scored for intensity as 1+ (weak staining/blush), 2+ (staining intensity between weak and intense), and 3+ (intense staining). Percentage is only given if the immuno-reactivity is not diffuse/present in all tumor cells. Specific comments regarding each section are listed. Columns in yellow highlight are scores of representative fields shown in Figure 7.

SUPPLEMENTAL METHODS

NHEJ assay. The NHEJ-DsRed system uses a lentiviral DNA cassette stably integrated into HCC1937 cells. The cassette contains two I-*Sce*I recognition sequences flanking an ATG initiation codon and has been described previously [1]. Forty-eight hours after HD-Ad infection, cells were infected with Ad-SceI and harvested at 0, 8, 16, or 24 h after Ad-SceI infection for DNA isolation. NHEJ events were determined by qPCR performed on an ABI 7900HT real-time PCR instrument using SYBR Green master mix (ABI). Relative NHEJ levels were determined after normalizing to β-actin. The PCR primers used were 5'-CACGAGACTAGCCTCGAGGTTT-3' and 5'-TTCCTCAAGTACGCGAAGTTC-3' for DsRed and 5'-TCACCCACACTGTGCCCATCTACGA-3' and 5'-GGTAACCGTTACTCGCCAAGGCGAC-3' for β-actin.

Trypan Blue/FACS cell-survival assay. HCC1937 cells were infected with HD-Ad vectors on 100 mm dishes. Forty-eight hours after infection, cells were trypsinized and 250,000 cells were seeded onto 60 mm dishes. Twenty-four hours after seeding, cells were irradiated with either 4 or 8 Gy or left unirradiated. Seven days after irradiation, the total number of cells in each dish were collected and re-suspended in equal volumes of media. Trypan Blue was added to the samples immediately prior to fluorescence-activated cell sorting (FACS) analysis as described [2].

Cell cycle analysis. HCC1937 cells were serum-starved, infected with HD-Ad vectors, and cells collected at 8, 16, 24, or 48 h after infection were fixed in 70% ethanol and cell-cycle distribution was analyzed by propidium iodide staining and FACS as described [3].

Scoring of human breast cancer tissue sections. Scoring of immunohistochemical stained sections was performed at Anatomic Pathology Research Services, Department of Pathology, Virginia Commonwealth University by a pathologist (M.O.I.) blinded to the study whose clinical specialty is breast pathology. Briefly, slides were reviewed using a Nikon Eclipse 80i light microscope and evaluated to ensure the presence of infiltrating and/or *in situ* carcinoma. Areas of fat necrosis or previous biopsy related changes were excluded from the evaluation as well as the edge of specimens because of edge effect.

SUPPLEMENTAL REFERENCES

1. Golding SE, Morgan RN, Adams BR, Hawkins AJ, Povirk LF, Valerie K. Pro-survival AKT and ERK signaling from EGFR and mutant EGFRvIII enhances DNA double-strand break repair in human glioma cells. Cancer Biol Ther. 2009; 8:730-738.

2. Golding SE, Rosenberg E, Valerie N, Hussaini I, Frigerio M, Cockcroft XF, Chong WY, Hummersone M, Rigoreau L, Menear KA, O'Connor MJ, Povirk LF, van Meter T, Valerie K. Improved ATM kinase inhibitor KU-60019 radiosensitizes glioma cells, compromises insulin, AKT and ERK prosurvival signaling, and inhibits migration and invasion. Mol Cancer Ther. 2009; 8:2894-2902.

3. Golding SE, Rosenberg E, Khalil A, McEwen A, Holmes M, Neill S, Povirk LF, Valerie K. Double strand break repair by homologous recombination is regulated by cell cycle-independent signaling via ATM in human glioma cells. J Biol Chem. 2004; 279:15402-15410.
